# Localising Loci underlying Complex Trait Variation Using Regional Genomic Relationship Mapping

**DOI:** 10.1371/journal.pone.0046501

**Published:** 2012-10-15

**Authors:** Yoshitaka Nagamine, Ricardo Pong-Wong, Pau Navarro, Veronique Vitart, Caroline Hayward, Igor Rudan, Harry Campbell, James Wilson, Sarah Wild, Andrew A. Hicks, Peter P. Pramstaller, Nicholas Hastie, Alan F. Wright, Chris S. Haley

**Affiliations:** 1 National Institute of Livestock and Grassland Science, Tsukuba, Japan; 2 The Roslin Institute and R(D)SVS, University of Edinburgh, Midlothian, United Kingdom; 3 MRC Human Genetics Unit, MRC Institute of Genetics and Molecular Medicine, University of Edinburgh, Western General Hospital, Edinburgh, United Kingdom; 4 Centre for Population Health Sciences, University of Edinburgh, Medical School, Edinburgh, United Kingdom; 5 Croatian Centre for Global Health, Faculty of Medicine, University of Split, Split, Croatia; 6 Institute of Genetic Medicine, European Academy Bozen/Bolzano (EURAC), Bolzano, Italy. Affiliated Institute of the University of Lübeck, Germany; 7 Department of Neurology, General Central Hospital, Bolzano, Italy; 8 Department of Neurology, University of Lübeck, Lübeck, Germany; University of Queensland, Australia

## Abstract

The limited proportion of complex trait variance identified in genome-wide association studies may reflect the limited power of single SNP analyses to detect either rare causative alleles or those of small effect. Motivated by studies that demonstrate that loci contributing to trait variation may contain a number of different alleles, we have developed an analytical approach termed Regional Genomic Relationship Mapping that, like linkage-based family methods, integrates variance contributed by founder gametes within a pedigree. This approach takes advantage of very distant (and unrecorded) relationships, and this greatly increases the power of the method, compared with traditional pedigree-based linkage analyses. By integrating variance contributed by founder gametes in the population, our approach provides an estimate of the Regional Heritability attributable to a small genomic region (e.g. 100 SNP window covering ca. 1 Mb of DNA in a 300000 SNP GWAS) and has the power to detect regions containing multiple alleles that individually contribute too little variance to be detectable by GWAS as well as regions with single common GWAS-detectable SNPs. We use genome-wide SNP array data to obtain both a genome-wide relationship matrix and regional relationship (“identity by state" or IBS) matrices for sequential regions across the genome. We then estimate a heritability for each region sequentially in our genome-wide scan. We demonstrate by simulation and with real data that, when compared to traditional (“individual SNP") GWAS, our method uncovers new loci that explain additional trait variation. We analysed data from three Southern European populations and from Orkney for exemplar traits – serum uric acid concentration and height. We show that regional heritability estimates are correlated with results from genome-wide association analysis but can capture more of the genetic variance segregating in the population and identify additional trait loci.

## Introduction

Despite the success of genome wide association studies (GWAS) in detecting new loci associated with complex traits, for most traits only a relatively low proportion of the total genetic variation has been localised. Although a variety of genetic mechanisms may contribute to this missing heritability [1], a significant component is likely to be the lack of power of GWAS to detect rare causative alleles that individually generate little SNP-associated variation but which collectively may contribute a substantial fraction of the heritability [2]. Thus even individual loci that contribute substantially to trait variation by dint of harbouring a number of functional alleles may be missed unless they contain at least one allele of sufficient effect and frequency to be identified in a GWAS.

To circumvent the drawbacks of single-SNP association analyses requires approaches that are more efficient at capturing the variance of individual rare causative alleles and are capable of accumulating the variance over all alleles at a locus. Appropriate linkage based analyses have some advantages over association analyses for detection of multiple rare variants at a locus because they integrate variance contributed by founder gametes within a pedigree, making no assumption about the association of individual gametes with particular causative alleles. However, pedigree-based linkage analyses lack power to detect true effects in the small nuclear families that typify a human population [3], although power to detect effects in extended pedigrees is substantially greater [4].

In this study we develop and demonstrate an approach to the analysis of genome-wide SNP data that integrates multiple allelic effects providing power to detect some loci that would be missed by standard analyses. This combines the ability of linkage analysis to integrate over all allelic effects at a locus with the ability of SNP based association analyses to capture variance across the whole population. To do this we use genome-wide SNP data to estimate the genetic relationships between all pairs of individuals in the population, both at the level of the whole genome and for each region within the genome. We then employ these relationships to estimate the trait variance contributed both by the genome as a whole (the genomic heritability) and by short regions of the genome (the regional heritability). The genomic heritability provides an estimate of the overall heritability but also controls for population structure. Studies by us and others have demonstrated that using the pedigree or genomic relationship matrix in a mixed model to estimate single SNP effects in pedigree structured data proves a powerful and unbiased analysis [5]–[7]. Thus inclusion of the genomic relationship means that the regional heritability is unbiased by overall population structure and hence provides a metric that indicates local genomic regions contributing to trait variation. To demonstrate this approach we have analysed simulated and real data from Southern European and Northern European populations based on exemplar traits – serum uric acid concentration and height. A mixed statistical model framework utilising restricted-maximum likelihood was used to estimate the heritability associated with each region of consecutive SNPs in the genome. The estimated heritability integrates genetic variation in that region and hence captures variance associated with the combination of common and rare variants segregating in the region. The analyses demonstrated that estimates from the regional heritability approach were very similar to those of single marker association analysis when two alleles segregate in a region. However when a cluster of functional variants segregate in a region the regional heritability approach captured more of the genetic variance segregating in the population, locating loci that would otherwise remain undetected.

## Results

### Analyses of simulated data

**Figure 1:**
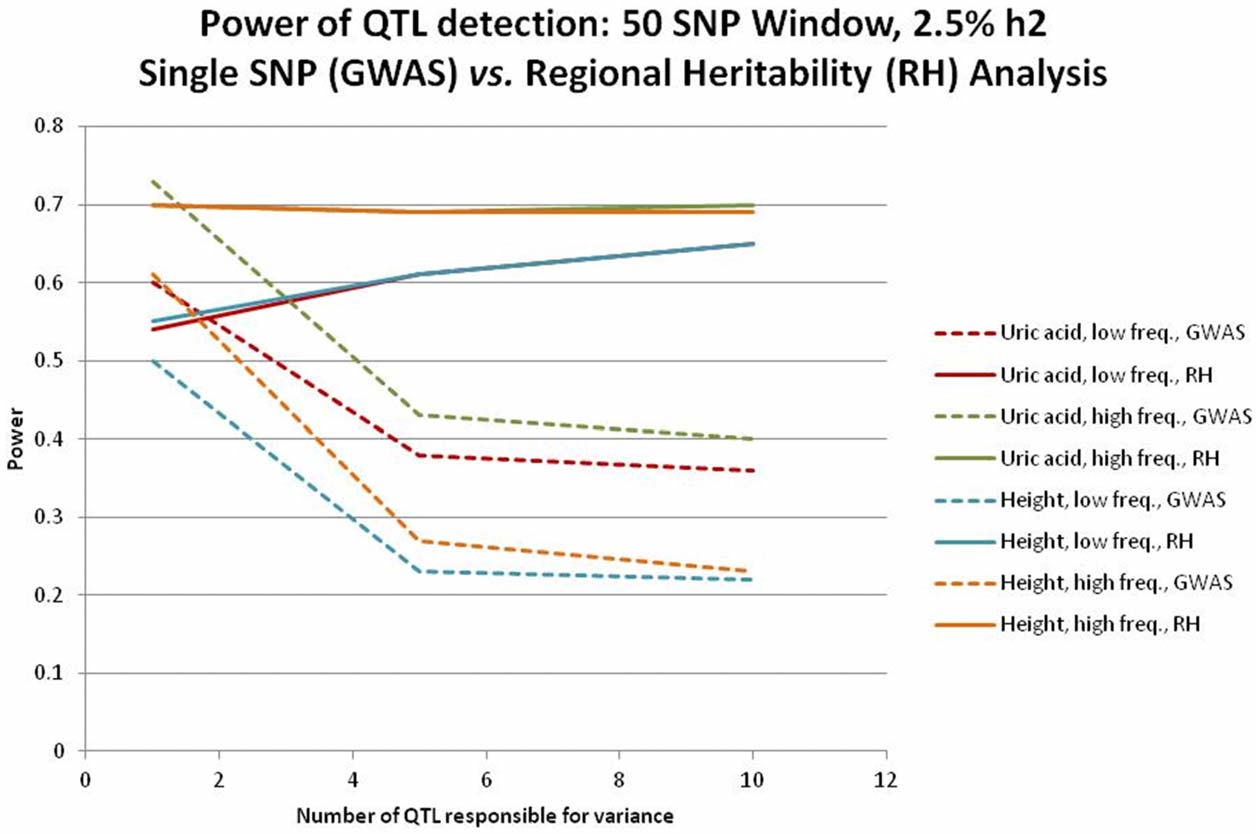
Power of GWAS and Regional Genomic Relationship Mapping in simulated data. Data were simulated based on real data by random selection of 1, 5 or 10 genotyped SNPs at either high or low MAF from a region of 50 SNPs. In each case the total simulated effects of the SNPs in a region contributed 2.5% to the heritability. The background trait heritability was either moderate at *circa* 30% as for serum uric acid concentration or high at *circa* 80% as for height. Power is the number of significant tests at the Bonferroni corrected threshold for either a GWAS of 275 k markers or a regional heritability analysis *circa* 11000 overlapping 50 SNP windows. Lines of the same colour represent results from the same simulated data produced by regional heritability analysis (solid) or by single marker analysis (dashed).

Our studies of overall genomic and regional genomic heritabilities are based on real data on serum uric acid concentration and height from a combined population of around 3000 individuals with marker data from *circa* 275000 autosomal SNPs. To explore the power of the regional approach compared to the standard single SNP GWAS we analysed the real data for each of a moderate heritability (uric acid) and a high heritability (height) trait in which an additional 2.5% additive genetic variance was simulated for each genomic region in turn. The additional variance was generated by simulating phenotypic effects associated with 1, 5, or 10 genotyped SNPs of either higher or lower MAF. The simulated data were analysed with the single SNP GWAS approach or via the regional heritability approach with windows of 50 adjacent markers. Power was estimated by comparison with the appropriate genome-wide significance threshold and is shown in Figure 1. For the standard single SNP GWAS (dashed lines) power to detect the simulated effects was highest when the regional effect was generated by a single SNP and fell substantially when 5 or 10 SNPs generated the variance. The power of standard single SNP GWAS was also greater when regional genetic variance was generated by the higher MAF SNP(s) and also greater for the lower heritability trait. In contrast, the most notable feature of the regional heritability approach (solid lines) was that its power was little affected by the number of SNPs that generated the regional genetic variance, actually increasing slightly when generated by more lower MAF SNPs. Also, the power of the regional heritability analyses was virtually unaffected by the level of the overall trait heritability, although power was reduced when the regional variance was generated by lower MAF SNPs. Overall, the only two situations in which genome-wide power to detect an effect was slightly greater with the single SNP GWAS was when the variance in a moderate heritability trait was generated by a single SNP of either higher or lower MAF. In all the remaining 22 combinations of parameters, power was greater for the regional heritability approach, with the difference always being substantial when trait variance was generated by multiple segregating alleles.

### Analyses of real population data on serum uric acid concentration and height

**Figure 2:**
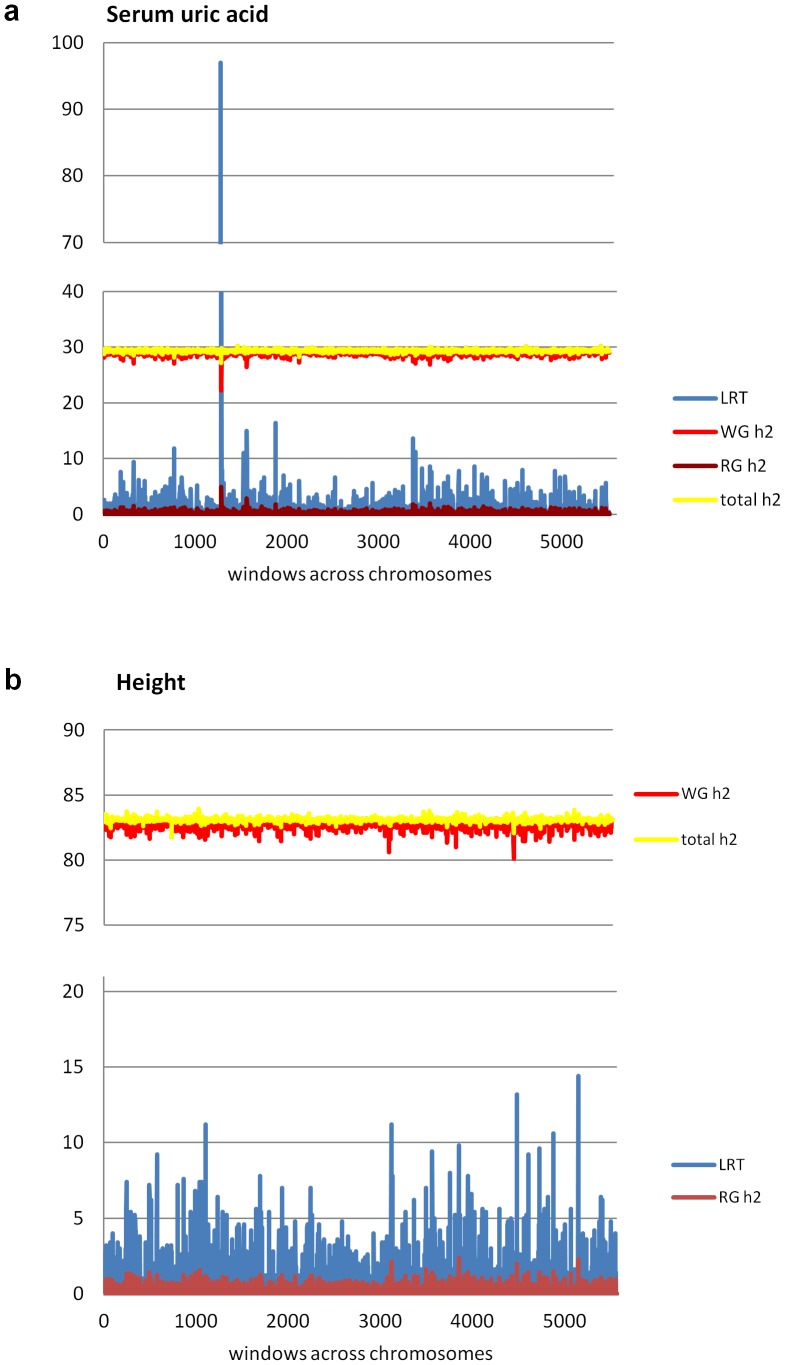
Results from genome-wide analyses of two traits. Plots show the likelihood ratio test (LRT) and regional, genomic and total heritabilities across the genome from analyses of data from Croatian and Italian populations: a) serum uric acid concentration and b) height. Vertical axis is the LRT and heritability (%) and horizontal axis is window number across the genome. RG h2, WG h2 and total h2 are regional heritability, residual whole genome heritability and total (sum of genomic and regional) heritability, respectively.

Figure 2 shows the estimated regional, genomic and total heritabilities and corresponding values for the likelihood ratio test (LRT) of the regional heritability across the genome for the real data on uric acid concentration and height in the combined analysis of population samples from Croatia and Italy (N = 3039). For uric acid concentration (Figure 2a) the total heritability averaged 29.30±0.15% across the genome and was less variable than the average regional (0.14±0.27%) and residual genomic (29.16±0.35%) heritabilities. The estimated correlation between the regional and the residual genomic heritabilities was −0.905 across all windows, indicating that a non-zero regional heritability drains genetic variance from the residual genomic heritability, whilst the overall heritability remains relatively constant. For serum uric acid concentration (Table 1), we show the results for the eight top regions with regional heritabilities estimated as >1%. We identified one region significant at the genome-wide level (p<0.05) with LRT more than 17.1 (which is the Bonferroni-corrected genome-wide significance threshold; window number 21 on chromosome 4). Four other regions were significant at a suggestive level, with a LRT of more than 11.4 for uric acid. Regional heritabilities varied from 1.22 to 4.93% for these regions. The highest regional heritability of 4.93% corresponded to the largest LRT 97.0 (window 21 on chromosome 4). For that window, the residual genomic heritability was 22.2% compared to the average of 29.2% across the genome.

**Table 1:** Regional heritability using single and multiple regional relationship matrices (100 SNPs) for serum uric acid.

Chromosome	Window number	SNP and position (bp) start end	LRT^1^	Single^2^h^2^	Multiple min h^2^ ^3^	Multiple max h^2^ ^4^	Average h^2^ ^5^
4	21	rs1282 rs4333176 8630658 10084378	97	4.93	4.44	4.58	4.53
5	277	rs248450 rs154111 150556960 151421588	16.4	1.75	1.68	1.92	1.80
4	301	rs8752 rs3733398 175649052 176792579	15	2.81	2.81	2.82	2.82
10	150	rs3740447 rs7900882 71977107 72550518	13.6	1.77	1.45	1.58	1.53
2	351	rs13404250 rs4666767 187824441 189475126	11.8	1.22	0.92	1.1	1.04
10	177	rs4933317 rs2642610 86006146 87051093	11.2	1.51	1.54	1.79	1.63
4	264	rs954368 rs17375199 154991226 156013670	11	1.33	0.9	1.07	1.01
1	329	rs3747636 rs12145634 202670282 203665187	9.4	1.49	1.2	1.48	1.39
Sum of regional h^2^				16.81	14.94	16.34	15.72
Genomic h^2^							13.58
Total h^2^							29.30

(LRT from window 21 on chromosome 4 and window 277 on chromosome 5 were significant (P<0.05) at the genomic level the remaining 6 windows were significant at the suggestive level).

1 Likelihood ratio test for regional heritability >0;

2 Estimated regional heritability in model with that single region and genomic effects;

3 Minimum heritability for that region from models with sets of 5 regional effects and genomic effect;

4 Maximum heritability for that region from models with sets of 5 regional effects and genomic effect;

5 Average heritability for that region over models with sets of 5 regional effects and genomic effect.

Positions are from the assembly build NCBI36/hg18.

For height (Figure 2b, Table 2), the total genetic heritability was averaged 83.1±0.09%, with more variable regional (0.14±0.25) and residual genomic (82.9±0.26) heritabilities. The ten most significant regions have regional heritabilities estimated as >1% and two regions reached the suggestive significance level (LRT>11.4), but no region reached the genome-wide significance level.

**Table 2:** Regional heritability using single and multiple regional relationship matrices (100 SNPs) for Height.

Chromosome	Window number	SNP and position (bp) start end	LRT^1^	Single h^2^ ^2^	Multiple min h^2^ ^3^	Multiple max h^4^ ^4^	Average h^2^ ^5^
19	27	rs1035458 rs7255203 12377150 13633030	14.4	2.28	2.22	2.38	2.29
15	31	rs1520954 rs12905733 35497445 36526590	13.2	1.96	1.84	2.06	1.94
3	222	rs9882269 rs12495327 115645413 116804048	11.2	1.12	0.97	1.03	1.01
9	160	rs10868320 rs868823 87357661 88409601	11.2	2.11	1.99	2.2	2.11
17	97	rs972872 rs9894487 51926412 52725682	10.6	1.48	1.36	1.53	1.45
12	61	rs11047882 rs1471506 25232444 26201501	9.8	1.59	1.17	1.63	1.40
16	111	rs7204751 rs987885 71637184 72250937	9.6	1.42	1.23	1.38	1.31
11	35	rs10765970 rs11022778 12568425 13347436	9.4	1.42	1.38	1.53	1.46
2	152	rs11126095 rs962856 66809407 67447307	9.2	1.24	1.07	1.3	1.15
15	155	rs745104 rs1002941 98316888 99060213	9.2	1.4	1.31	1.31	1.31
Sum of regional h^2^				16.02	14.54	16.35	15.43
Genomic h^2^							67.67
Total h^2^							83.1

(LRT from all windows were significant at suggestive level).

1 Likelihood ratio test for regional heritability >0;

2 Estimated regional heritability in model with that single region and genomic effects;

3 Minimum heritability for that region from models with sets of 5 regional effects and genomic effect;

4 Maximum heritability for that region from models with sets of 5 regional effects and genomic effect;

5 Average heritability for that region over models with sets of 5 regional effects and genomic effect.

We explored the overall estimated variance explained by the regions significant at the suggestive level. For uric acid concentration the sum of the five suggestively-significant regional heritabilities when each was estimated singly as a separate region (Table 1) was 12.48%. This sum included only the single highest value where adjacent windows reached the suggestive level. To assess the effect of non-independence between regions we fitted a model including all five regional genomic relationship matrices simultaneously and estimated a total variance explained of 11.72%. Thus an estimated 40% of the total genetic variance in uric acid concentration of 29% was explained by these 5 regions together. For height, the contribution of the two suggestive regions to the total variance is 4.3%, representing approximately 5% of the total genetic variance estimated at 83.1%.

### Comparison of regional estimates and GWAS results from real data

**Figure 3:**
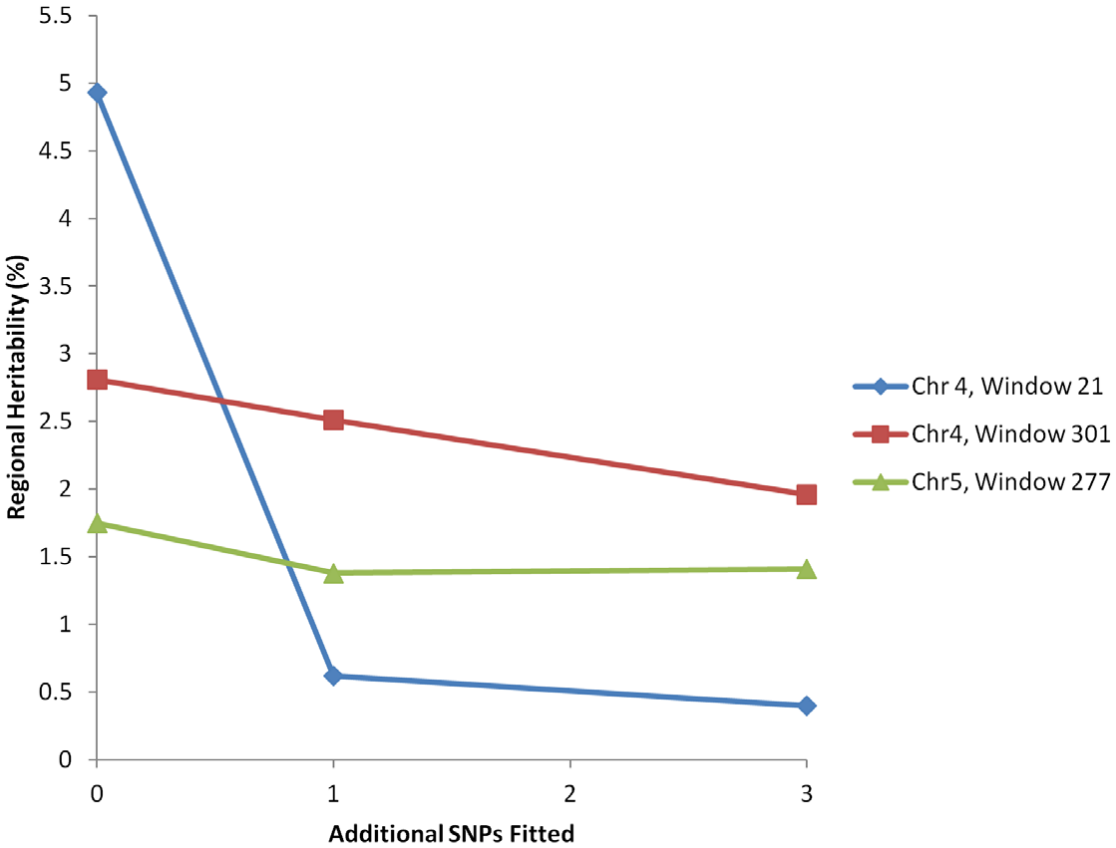
Regional heritabilities for uric acid concentration for three most significant windows. Heritabilities are estimated in a model with both regional and genomic genetic effects using all 100 SNPs in the window to derive regional relationships and fitting 0, 1 or 3 SNPs with the highest –Log_10_ P value from the GWAS in that window as covariates in the analysis.

Standard single SNP GWAS of the real data were performed using GenABEL. In the analysis of combined data from Croatia and Italy, for uric acid concentration 14 SNPs were significant (-Log_10_P>6.7) at the genome-wide level after Bonferroni correction with all of these SNPs coming from window 21 on chromosome 4. There were no SNPs significant at the genome-wide level for height for these data. In order to explore the extent to which the regional heritability could explain more variance than that associated with individual SNPs in the real data we estimated the regional heritability in data from Croatia and Italy in a model in which one or more SNPs have been included as covariates. This allows estimation of the regional heritability not explained by the significant SNPs or by SNPs in LD with them. We explored this approach for the three windows with the largest LRT for uric acid concentration (chromosome 4, windows 21 and 301 and chromosome 5, window 277). For each window we identified the three most significant individual SNPs from the single SNP GWAS and used these as covariates in the analysis. The estimated regional heritabilities for these three regions fitting either 0 or 1 or 3 most significant SNPs are shown in Figure 3. For chromosome 4, window 21, fitting the single most significant SNP reduced the regional heritability estimate from 4.93% to 0.4% - a reduction of over 90% in the estimate. For the other two regions fitting the most significant single SNP reduced the estimated regional heritability by only 11% and 21% for the two regions, respectively. Fitting jointly all three SNPs reduced the estimated regional heritabilities by 30% and by 20%, for these latter two regions respectively. Thus in the first of these three regions single SNP analysis captures much of the variance explained by the regional heritability approach, but for the latter two regions the regional heritability approach captures variance not readily captured by the SNPs individually.

**Figure 4:**
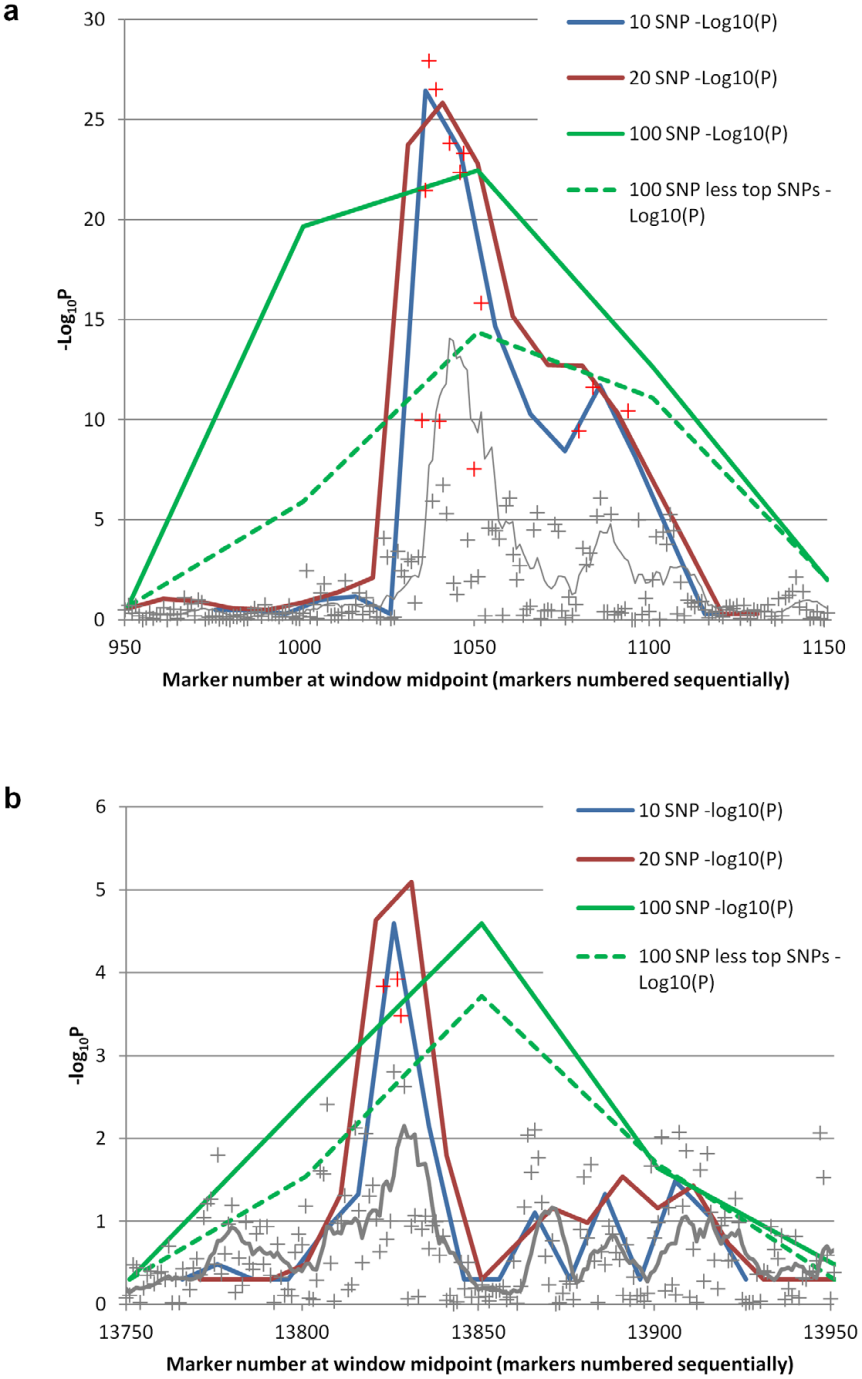
Comparisons of regional heritability and GWAS results for two most significant windows for uric acid concentration. Comparisons shown on –log_10_ P basis by conversion of likelihood ratio test (LRT) statistic assuming is distribution is a mixture of half χ_1_^2^ and half zero. Lines indicate results for regional heritability for three window sizes (100 SNP = green; 20 SNP = red; 10 SNP = blue). Crosses show results for individual SNPs in GWAS with grey line showing moving average of 10 adjacent SNPs. **a**) Results for chromosome 4, window 21 (SLC2A9 region). Additional dashed green line is result for 100 SNP window with relationships estimated omitting 14 most significant individual SNPs (in red). **b**) Results for chromosome 5, window 277. Additional dashed green line is result for 100 SNP window with relationships estimated omitting 3 most significant individual SNPs (indicated by red crosses).

Use of 100 SNP windows to estimate regional heritabilities integrates information over approximately 1 Mb regions (Table 1) and limits issues associated with multiple testing. However smaller windows can be used to improve mapping resolution and throw light on the genetic architecture in an associated region. Figure 4a shows a comparison of the results for 10, 20 and 100 SNP windows for the two most significant regions for uric acid concentration in the combined data from Croatia and Italy. For both regions the use of shorter windows improves the apparent resolution without reducing the magnitude of the test statistic. Consistent with our earlier results, for the chromosome 4, window 21 results, the most significant individual SNPs from the GWAS analysis have slightly higher test statistics (accompanied by a higher level of multiple testing) than that from the regional heritability approach but the average of 10 adjacent SNPs from the GWAS is substantially worse than the 10 SNP regional heritability window. The result from window 277 on chromosome 5 (Figure 4b) contrasts with this in that no single SNPs have comparable test statistics with the regional heritability approach and the average of 10 adjacent single SNPs is somewhat lower. This again illustrates the improved capture of genetic information from the regional heritability approach.

We then explored the overlap between regions detected in the regional heritability analysis of data from Croatia and Italy and single associated SNPs detected in large meta-analyses. One of the largest meta-analyses yet published is that for height, which based on data from more than 183000 individuals identified 180 loci [8]. Of the ten regions we report in Table 2, four contain one of the 180 reported loci. The ten regions in Table 2 span 8.3 Mb, thus assuming a genome of 3 Gb the probability of 4 SNPs out of 180 falling into this 8.3 MB by chance can be estimated to be 0.0017, thus these regions are significantly enriched for SNPs detected in the meta-analyses. We also compared the reported 180 SNPs and the most significant 10 SNPs from a standard single SNP GWAS in our data, allowing a 0.5 Mb window each side of each of these 10 most significant SNPs. Only one locus from the 180 loci [8] was located within the total span of 10 Mb around the 10 most significant SNPs, which is not significantly more than would be expected by chance. No meta-analysis of comparable scale has yet been published for uric acid concentration, but a recent large-scale analysis identified 11 loci associated with gout and/or serum uric acid levels, most of which had been identified in previous analyses [9]. In our analysis of uric acid concentration, the top eight regions identified each explaining more than 1% of the variance overlapped with only one of the loci identified in the meta-analysis. Although even this level of overlap is unlikely to occur by chance (p = 0.032), the most significant region contains the SLC2A9 locus which contains SNPs significant in our single SNP GWAS of these data. It is also interesting to note that only the SLC2A9 region in the meta analysis is estimated to explain >1% of the trait variance (at 3.5% overall), the other detected associations explain between 0.1 and 0.6% of the trait variance. Such small effects would be unlikely on their own to be detectable in our regional heritability analysis of modest size unless other alleles segregating in the same regions boosted the regional variance substantially.

**Figure 5:**
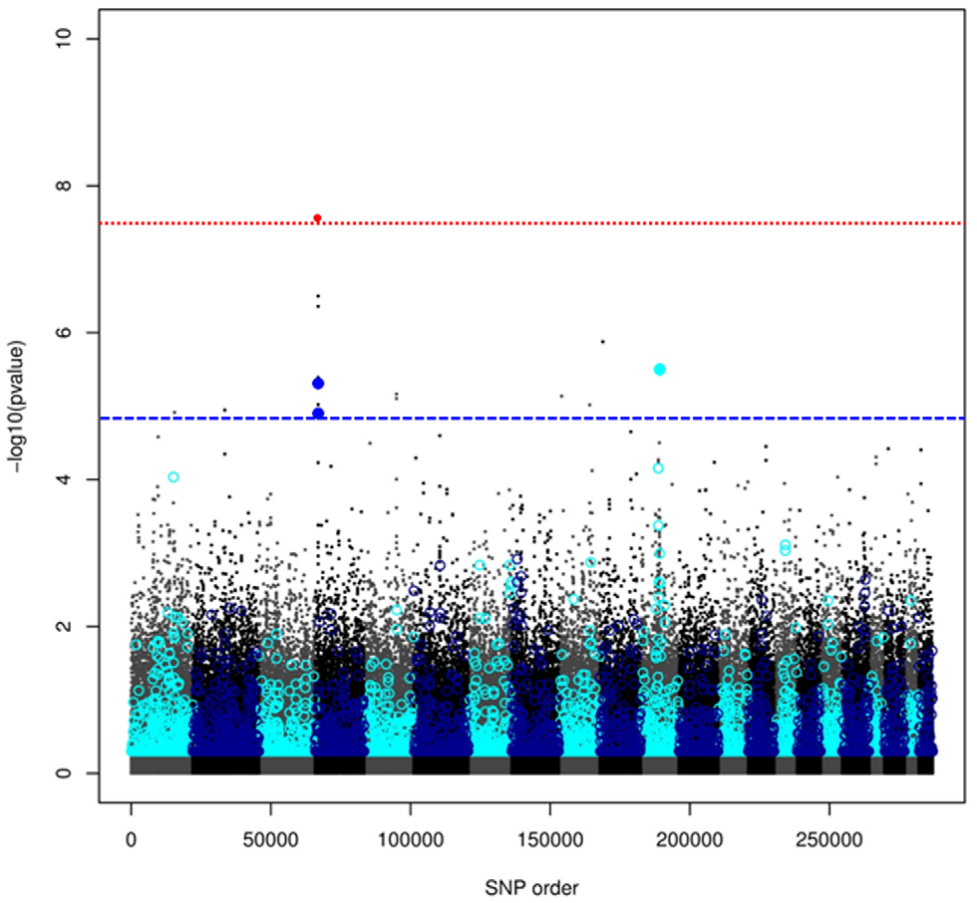
Comparison of single marker analysis and regional heritability analyses of serum uric acid concentration in a population from Orkney. -log P values are plotted against position in the genome. Points represent individual markers and circles results from regional heritability analysis with 100 marker windows. Genome-wide significance thresholds are represented by dashed lines (red for single marker analysis, blue for regional heritability analysis). Alternating shades represent the separate chromosomes. Results surpassing the genome-wide significance threshold are solid red for single marker analysis and solid blue for regional heritability analysis.

Finally we compared the results of regional heritability analysis of serum uric acid concentration of the small sample from Orkney with standard single SNP GWAS analysis of the same data and loci identified in previous analyses [9]. The results of the two analyses are shown in Figure 5. The standard single-marker GWAS analysis using GenABEL [21] identified one SNP significant at the genome-wide level of significance (-log_10_P>7.3) located at the SLC2A9 locus on chromosome 4, two further SNPs were significant at the suggestive level (-log_10_P>6.0) at the same locus. The total genomic heritability was slightly high than in the Southern European populations at 40%. The regional heritability analysis identified three regions above the genome-wide threshold (LRT>17.1, equivalent to -log_10_P>4.75). Two of these were overlapping regions at the SCL2A9 locus, the third on chromosome 11 (71548134–72664686 bp) does not correspond to any reported associated loci or obvious candidate loci. Two further regions were significant at the suggestive level (LRT>11.4; -log_10_P>3.44). That on chromosome 1 (179941514–180860387 bp) does not correspond to any known associated loci. However, the higher of these two suggestive regions on chromosome 11 (63640603–65051406 bp) spans the SLC22A11 and SLC22A12 loci that have been previously identified as associated with serum uric acid concentration [9]. The estimated variance explained by this latter region in our analysis is 6.7%, whereas the sum of the variance explained by the SNPs in SLC22A11 and SLC22A12 as estimated in the meta-analysis was 0.32% [9] again suggesting that the regional heritability analysis may capture additional variance not detected by single marker analyses.

### Permutation

**Figure 6:**
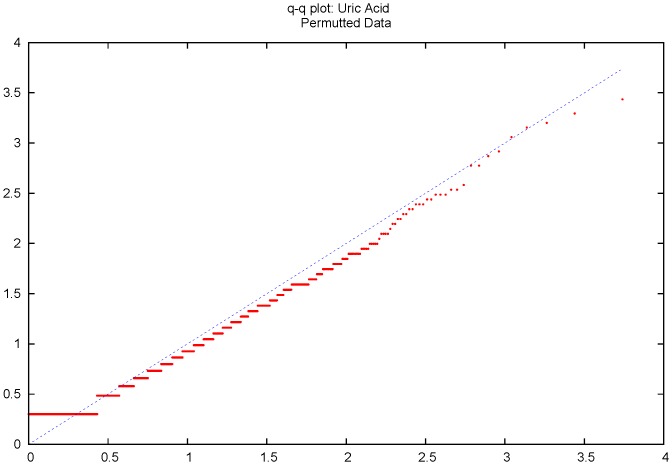
Q-Q plot of distribution of observed against expected –log_10_ P values from permuted data. Observed values are calculated assuming the LRT is distributed as 1/2χ^2^(0)+1/2χ^2^(1). The dotted line shows the slope of unity.

To test the null hypothesis that the regional variance component was zero we used the standard assumption that the asymptotic distribution of the likelihood ratio test will be distributed as 1/2χ^2^(0)+1/2χ^2^(1). This distribution arises when the true regional variance component is zero with half the estimates across regions being greater than zero and half bounded to zero by the estimation procedure [10], [11]. We explored the actual distribution of the test statistic using permutation. Figure 6 shows the resulting Q-Q plot of the observed regional tests against those expected assuming the test statistic distribution of 1/2χ^2^(0)+1/2χ^2^(1). The observed distribution of –log_10_ p values is parallel to, but slightly below that expected. This suggests that the use of the significance threshold based on an expectation that the test statistics follow this distribution will be slightly more stringent than anticipated, in consequence reducing the power of the test of the regional heritability slightly. On this basis using permutation to set the threshold for the regional heritability analysis would increase power to detect effects compare to the results shown here.

## Discussion

In this study we introduce an approach to the analysis of complex trait data from a GWAS study that is capable of localising some of the variation that has escaped detection by standard GWAS analyses. By utilising both whole genome and regional relationship matrices derived from high-density SNP data, it is possible to estimate the variance attributable to short regions of the genome. In estimating between gamete variance within a region, the approach has the ability to integrate the variance over all variants segregating in the region. It thus provides an estimate of variance attributable to a region that might be due to several or many segregating alleles at a single locus and/or the joint effect of closely located loci.

We have applied this approach to two separate traits, serum uric acid concentration and height, recorded in over 3000 individuals from Croatia and Italy and 854 from Orkney.. In the analyses of serum uric acid concentration from Croatia and Italy, the approach fully captures the variance attributable to the known effects of SLC2A9 on chromosome 4. With four other regions significant at the suggestive level, the total trait variance explained is around 12%, or approximately 40% of the total genetic variance, although these estimates are likely to be inflated by selection of the most significant windows and hence the “winner's curse" [12]. For height, in these data, we identify 10 windows which individually explain between 1.01 and 2.29% of the trait variance (Table 2). The joint estimate of the variance is around 19% of the total genetic variance, again probably inflated by the “winner's curse". The 10 regions identified are significantly enriched for SNPs associated with height identified in the meta-analysis of GWAS data [8]. This is in contrast to windows flanking the 10 most significant SNPs identified in standard single-SNP analysis of our data. The analyses of serum uric acid concentration in the small data set from Orkney also pick out the region associated with SLC2A9. Of the other three regions significant at the suggestive level or above one contains two loci previously identified by a meta-analysis as associated with the trait. The estimated variance explained by this latter region is substantially greater than that estimated in the previous meta-analysis. This estimate might be inflated by the “winner's curse" but it could also indicate additional variance due to a rare allele or alleles that are a potential characteristic of such population isolates.

As a further insight on relative power of our approach to detect effects of a given size, we can consider the comparison with the standard SNP by SNP analysis. The two analyses use to some extent different sources of information and their relative values will depend upon the true genetic architecture of the trait. Thus, if a single SNP is in complete association with the causative variant (i.e. r^2^ = 1) then the standard single-SNP based association analysis should be the most powerful analytical approach but, when association with any single SNP is incomplete and there may be several causative alleles, some of which are potentially rare, an approach such as the one used here may be advantageous. Consider our analyses of the three most significant regions affecting uric acid concentration. For window 21 on chromosome 4 we found that including a single SNP in the regional heritability analyses almost completely abolished the regional heritability and associated LRT. This suggests that the causative variant (or perhaps haplotype) is strongly associated with this single SNP with limited additional variance in this region. In contrast, in the other two regions we explored no single SNPs had previously been identified and fitting the single most significant SNP had only a limited effect on the estimated regional heritability and associated LRT. This suggests that these regions contain one or more causative variants that cannot be explained by association with a single SNP but can be detected by the composite measure that the regional heritability estimate represents. In the most significant region chromosome 4, window 21, the SLC2A9 locus is well established as the likely candidate [13]. The significant region for serum uric acid levels in chromosome 5 harbours SLC36A2, an amino acid (glycine- proline)/proton transporter expressed in the kidney, specifically in the S1 segment of the most proximal part of the convoluted tubule adjacent to glomerulus [14], which is a plausible biological candidate. Replication of this regional effect would suggest the segregation of one or more alleles in this population that are not readily detected by single marker GWAS analyses.

The conclusions about the relative performance of methods based on the analyses of real data are supported by those derived from the simulated data. We show that even in the most favourable situation for a single-SNP based GWAS when trait variation is generated by a single biallelic locus, the GWAS and regional heritability approaches have similar power. The power to detect effects by a single-SNP GWAS drops when there is more than a single locus in a region contributing the same total variation, but this is not the case for the regional heritability approach. Hence the regional heritability approach has a substantial advantage in power over the single-SNP GWAS when several loci in a region contribute trait variation. We simulate trait associated loci using actual genotyped SNPs which are then not used in the analysis. This then generates linkage disequilibrium representative of the population and by using each window across the genome we sample the whole range of relevant genetic architectures.

We have shown that the regional heritability is moderately correlated with the average test statistic from a standard association analysis of the individual SNP within the region, thus the approach is using some of the same information. However, by using information from all SNPs in a region, information is incorporated from across wider relationships, including all those for which pedigree relationships are not recorded because the most recent common ancestor occurs before available records. Use of such information has the effect of increasing family sizes beyond those recorded and hence increasing power to detect regional effects. In addition, the larger number of meioses that separates more distant relationships potentially provides more precise localisation of effects than that obtained in traditional pedigree-based linkage analysis.

As with all analyses, the power to detect a significant regional heritability will be strongly influenced by sample size. With our sample the empirical results suggest that regions which are estimated to explain a little over 1% of the variance can be detected at the suggestive level, whereas regions estimated to explain around 2% of the trait variance may be significant at the genome wide level. This is borne out by the simulation studies where we demonstrate up to 70% power to detect effects contributing 2.5% of the trait variance. We would expect the LRT test statistic to scale approximately linearly with both sample size (for a given structure of the sample) and variance explained [15], [16]. Thus if the sample size was to be quadrupled to 12 000 individuals in a similar structure, regional heritabilities of around 0.5% estimated effect might be expected to be significant at the genome-wide level. A second relevant factor to consider is the population structure itself. The samples analysed here derive from population isolates, which, although not inbred, are more closely related than would be individuals sampled from a large panmictic population. The approach used here where the main analysis uses 100 SNP windows is likely to be more powerful in populations such as the ones we have used where haplotypes of such a size are likely to be conserved intact between even distantly relatives. In less related populations, shorter windows may better capture the more distant relationships. Such populations would have the advantage that the whole genomic relationship matrix will be less correlated with regional relationship matrices, which should make regional effects easier to disentangle from those of the whole genome. On the other hand the greater number of analyses increases computational load and the multiple testing penalty in setting a genome wide significance threshold. The optimum balance of close and distant relationships and the size of the window in relation to the population structure for dissection of regional genomic effects from those of the whole genome remains to be determined, and will partly be dependent on population structure and genetic architecture.

Estimation of the variance associated with regions of the genome has been explored a number of times, initially using combined information on markers and pedigree [17] and more recently using dense marker data in the absence of pedigree information [2], [18]. Our approach is related to those of Hayes *et al.* [18] and Yang *et al.* [2], but we show here how we can extract meaningful regional results based on relationships estimated with as few as 10 adjacent markers. It is also interesting to note that the total genomic heritabilities estimated here are close to those reported for pedigree based analyses of the same traits in contrast to the lower estimated heritability for height from Yang *et al.* [2]. This is likely to be due to the fact that we explicitly make use of both close and distant genetic relationships and hence exploit within pedigree associations in addition to the population level LD exploited in the more distant relationships used by Yang *et al.* [2]. We note also the recent discussion on the potential contribution of gene interactions to estimates of additive genetic variance [19]. We anticipate that whilst our estimates of the overall genomic heritability may be inflated by gene interactions, estimates of regional heritability will only incorporate variance due to local interactions within the region. These may contribute to an improved ability to detect variance associated with the local region over and above that we have demonstrated when regional effects are due to only the additive effects of multiple segregating alleles.

The approach to the analysis of genome-wide SNP data that we introduce and explore has the potential to capture some of the heritable variance that escapes the standard SNP by SNP analysis. The use of regional windows and estimation of variance in a mixed model framework integrates over the gametic variance in a region and escapes from reliance on the association between single causative alleles and single SNP alleles. It thus has the ability to integrate effects over several causative variants providing a joint estimate of the combined effects of common and rare variants in a region. The example analyses we present here suggest that regions known to harbour effects large enough to be detected by standard SNP by SNP analyses may yield some additional variance when analysed by this approach. Furthermore, regions where no single associated SNP has a large enough effect to be detected as significant at the genome wide level may explain sufficient variance to be detected by this approach. The analytical approach we present here is one way in which it may be possible to pin down some of the variance undetected by SNP-by-SNP analyses to particular regions, pathways and loci.

## Methods

### Ethics Statement

The Croatian cohorts were recruited from the island of Vis and the island of Korcula respectively, both approved by the Ethical Committee of the Medical School, University of Zagreb and the Multi-Centre Research Ethics Committee for Scotland. The Italian MICROS cohort was recruited from the villages in South Tyrol and approved by the ethical committee of the Autonomous Province of Bolzano. The ORCADES study was approved by the NHS Orkney Research Ethics Committee and the North of Scotland REC. All participants gave written informed consent.

#### Mixed model using whole genomic and regional genomic relationship matrices

We used a mixed model including the fixed effects of sex, population, village and age and random additive genetic effects which were divided into two parts, regional genomic and residual whole genomic additive genetic effects. The whole genomic additive effect was estimated by using all SNPs to construct the whole genomic relationship matrix. The regional genomic additive effect was estimated from a regional genomic relationship matrix constructed from 100 adjacent SNPs from each region. The same whole genomic relationship matrix was used in the analysis of all regions (i.e. the markers for the region under analysis were not removed), any consequential correlation generated between whole genomic and regional relationships would be very small and would in any event reduce the likelihood of detecting a regional effect. Genomic kinship f_ij_ between individual i and j using identity by state (IBS) is used where:
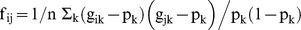
where g_ik_ (g_jk_) is the genotype of the i-th (j-th) person at the k-th SNP (coded 0, ½, 1 for rare allele homozygote, heterozygote and common homozygote, respectively) [20], [21]. The frequency p_k_ is for the major allele and n is the number of SNPs. For relationship matrices in mixed model equations, 2f_ij_ is used for the off-diagonal elements between individuals and the diagonal elements are one plus the inbreeding coefficient [20]. We followed a two-step method described in George et al. [22] for the variance component analysis using ASReml (VSN International, 2002). The mixed model is as follows:
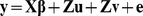


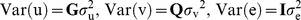
where the vector **y** represents the phenotypic values, **X** is the design matrix for fixed effects, and **Z** is the design matrix for random effects. The remaining vectors are, **u**: whole genomic additive genetic effect, **v**: regional genomic additive genetic effect, **e**: residual, and **β**: fixed effects. Adjustments for sex and regression on age were used for height and sex and regression on age within sex were used for uric acid. Matrices **G** and **I** are a whole genomic relationship matrix using all SNP for whole genomic additive effect and a unit matrix for residuals, respectively. **Q** is regional genomic relationship matrix obtained using 100 SNPs for regional genomic additive effect. Whole genomic, regional genomic and residual variances are σ^2^_u_ σ _v_^2^ and σ^2^_e_, respectively. Phenotypic variance, σ^2^_p_, is σ^2^_u_+σ _v_^2^+σ^2^_e_. Whole genomic heritability and regional heritability are h_u_^2^ ( = σ^2^_u_/σ^2^_p_), and h_v_^2^( = σ^2^_v_/σ^2^_p_), respectively. To confirm that fitting fixed effects for population and the whole genomic relationship matrix account adequately for population structure and any other stratification we also repeated the analyses including the first 10 principal components derived from analysis of the genotype data. This made only trivial difference to the test statistics for regional effects (not shown).

#### Test for significant regional variance

To test for the presence of regional variance against the null hypothesis (no regional variance) at a test region (window), the likelihood ratio (LR) test statistic LRT = −2ln(L_0_/L_1_) was calculated, where L_0_ and L_1_ represent the respective likelihood values under the hypothesis of either the absence (H_0_) or presence (H_1_) of regional variance. A window size of 100 SNPs was used to construct a regional relationship matrix and the window was shifted every 50 SNPs. Therefore, first, second and third regional matrix, for example, used from 1^st^ to 100^th^, 51^st^ to 150^th^ and 101^th^ to 200^th^ SNPs. In total, 5511 windows were tested across chromosomes. We applied a Bonferroni corrected threshold to test significant regions.

Additionally molecular maker score (mms [23]) and SNP genotypic values were also calculated by using the R package GenABEL [21]. The SNP effects were estimated after adjusting for fixed effects and genomic additive effects using whole genomic relationship.

#### Permutation and simulation

To explore the distribution of the test statistic under the null hypothesis of no regional effect (zero regional heritability) we used permutation. To implement this we permuted individuals against the regional relationship matrix whilst leaving intact the actual relationship between individuals, their phenotypes and the genomic relationship matrix. This will generate a population in which the overall trait heritability remains intact, but there are no real regional heritabilities. Permutation is carried out separately for each region on the genome (hence all regions are uncorrelated) and the subsequent scan of the whole genome simulated the distribution of test statistics under the null hypothesis.

We use simulation to explore the power of the approach under different genetic models and in comparison with a standard single SNP GWAS of the data. The genome was analysed as a series of overlapping 50 SNP windows as in the analyses of the real data. For every window, data were simulated based on the actual SNP genotypes. The SNPs were allocated to one of two groups, the 25 SNPs with the highest MAF and the 25 with the lowest MAF. One, five or ten SNPs were randomly selected from the high MAF group and similarly 1, 5 or 10 SNPs were selected from the low MAF group, thus six separate simulations for each of two traits were performed for each 50 SNP region in the genome. The selected SNPs were removed from the marker SNP set and used to generate additional trait variance. An additive effect was added to the actual trait value of an individual according to the genotype or genotypes of the selected SNP(s). The effects were scaled such that the total regional variance added was equivalent to 2.5% of the total trait variance in all situations (i.e. the same regional variance was added to the real data whether 1, 5, or 10 SNPs of high of low MAF were used for the simulation). The simulated regional effects were added to the real trait values for uric acid concentration or height. Thus in summary we simulated 2.5% additional trait variance generated by 1, 5, or 10 loci with high or low MAF for a moderate heritability trait (uric acid concentration) and a high heritability trait (height) for each of 11022 overlapping 50 SNP windows in the genome. Each simulated set of data was analysed either by a single marker analysis adjusting for the total genomic relationship between individuals or using the total genomic and regional relationships to estimate a regional heritability (methods as described above). Models were tested against the appropriate genome-wide significance thresholds assuming 5511 (11022/2) independent tests of the regional heritability or 275 k SNPs as appropriate. The markers randomly selected to become simulated QTL were not used in any analyses, hence the regional heritability analyses were based on windows of 49, 45 or 40 markers (for 1, 5 and 10 simulated QTL, respectively).

#### Study samples and genotyping

Measurements of serum uric acid concentration and height from unselected populations from Croatia and South Tyrol (Italy) were combined in the first analyses and data on serum uric acid concentration from the ORCADES study in Orkney were used in the second analysis. The Croatian data were from two Dalmatian islands, Vis and Korcula, and the data from South Tyrol were from three isolated villages located in the south of the region. The number of phenotypic records for serum uric acid was 3039 in the first analyses (1807 from Croatia and 1232 from South Tyrol) and 854 from Orkney in the second analysis, and was 2979 in the combined analysis for height (1791 from Croatia and 1188 from South Tyrol). Average age (sd) of subjects was 51.9 (16.3) and varied from 18 to 98.

We used 282,415, 302,507 and 286,959 autosomal SNPs (Illumina Human Hap300 for Vis, South Tyrol and Orkney and Illumina CNV370 for Korcula) from the Croatian, South Tyrol and Orkney data, respectively. These SNPs had passed our quality control protocol, that discards SNPs with minor allele frequency <0.02, out of Hardy-Weinberg equilibrium (p value<10^−6^) or call rate<0.98 and individuals with call rate <0.97. In total, 275,564 autosomal SNPs were common to both the Croatian and South Tyrol samples (comprising 3110 individuals, 1822 from Croatia and 1288 from South Tyrol) and were used in our first analysis. Further details on these data have been reported in previous papers [13], [24], [25].
